# Supplementary material for: Higher Risk of Recurrence in Patients Treated for Head and Neck Cancer with Low BMI and Elevated Levels of C-Reactive Protein
Source: Cancers (Basel). 2022 Oct 21;14(20):5161. doi: 10.3390/cancers14205161 (PMC9600233; doi:10.3390/cancers14205161)
Supplement: Supplementary file 1 [file cancers-14-05161-s001.zip › cancers-1908682-supplementary.pdf]

Supplementary Table S1. Treatment groups.

**Treatment**	**N**
Surgery only	30
Radiotherapy only	90
Preoperative radiotherapy + surgery	6
Surgery+ postoperative radiotherapy	54
Chemoradiation only	61
Preoperative chemoradiation+ surgery	3
Surgery + postoperative chemoradiation	11
Radiotherapy + cetuximab only	15
Surgery + postoperative radiotherapy + cetuximab	1
Preoperative radiotherapy + cetuximab + postop surgery	1
